# Parallel recovery of chromatin accessibility and gene expression dynamics from frozen human regulatory T cells

**DOI:** 10.1038/s41598-023-32256-6

**Published:** 2023-04-04

**Authors:** Ying Y. Wong, Jessica E. Harbison, Christopher M. Hope, Batjargal Gundsambuu, Katherine A. Brown, Soon W. Wong, Cheryl Y Brown, Jennifer J. Couper, Jimmy Breen, Ning Liu, Stephen M. Pederson, Maren Köhne, Kathrin Klee, Joachim Schultze, Marc Beyer, Timothy Sadlon, Simon C. Barry

**Affiliations:** 1 Robinson Research Institute, University of Adelaide, Adelaide, Australia; 2 German Center for Neurodegenerative Diseases, University of Bonn, Bonn, Germany; 3 Women’s and Children’s Hospital, North Adelaide, Australia

**Keywords:** Immunology, Molecular biology, Medical research

## Abstract

Epigenetic features such as DNA accessibility dictate transcriptional regulation in a cell type- and cell state- specific manner, and mapping this in health vs. disease in clinically relevant material is opening the door to new mechanistic insights and new targets for therapy. Assay for Transposase Accessible Chromatin Sequencing (ATAC-seq) allows chromatin accessibility profiling from low cell input, making it tractable on rare cell populations, such as regulatory T (Treg) cells. However, little is known about the compatibility of the assay with cryopreserved rare cell populations. Here we demonstrate the robustness of an ATAC-seq protocol comparing primary Treg cells recovered from fresh or cryopreserved PBMC samples, in the steady state and in response to stimulation. We extend this method to explore the feasibility of conducting simultaneous quantitation of chromatin accessibility and transcriptome from a single aliquot of 50,000 cryopreserved Treg cells. Profiling of chromatin accessibility and gene expression in parallel within the same pool of cells controls for cellular heterogeneity and is particularly beneficial when constrained by limited input material. Overall, we observed a high correlation of accessibility patterns and transcription factor dynamics between fresh and cryopreserved samples. Furthermore, highly similar transcriptomic profiles were obtained from whole cells and from the supernatants recovered from ATAC-seq reactions. We highlight the feasibility of applying these techniques to profile the epigenomic landscape of cells recovered from cryopreservation biorepositories.

## Introduction

Gene expression is regulated by the binding of transcription factors (TFs) to the proximal promoters and distal regulatory elements such as enhancers. Chromatin structure has a major influence on gene expression by controlling the access of TFs to binding sites in these enhancers and promoters^1^. Localised patterns of epigenetic modification of chromatin, such as histone modification, DNA methylation and nucleosome remodelling correlate with enhancer activity, TF binding and regulation of transcription. Chromatin accessibility relates to the degree to which proteins are able to physically interact with chromatin, and is strongly influenced by epigenetic modifications, nucleosome positioning and chromatin binding proteins that restrict access to the DNA^2^. More accessible or open chromatin is associated with transcriptional active regions and regulatory elements, while closed chromatin is associated with silent or repressed regions. For example, although accessible DNA makes up ~ 2–3% of the genome it captures more than 90% of the regions occupied by TFs assayed in the ENCODE project^2–4^. Chromatin accessibility is usually determined experimentally by the susceptibility of chromatin to enzymatic methylation or cleavage. However, conventional chromatin profiling methods such as DNase-seq requires millions of cells as the input requirement and involves complex library preparation workflow. Assay of Transposase Accessible Chromatin with high throughput sequencing (ATAC-seq) is a relatively new genome-wide approach that simultaneously probes chromatin structure (accessibility and nucleosome positioning) and importantly, TF binding at high resolution with lower input requirement than other techniques^5,6^. The development of ATAC-seq has made it possible to quantify chromatin accessibility using a much lower number of cells, therefore making it amenable to the clinical setting and rare cell populations of interest. The measurement of chromatin accessibility at single cell resolution has also become possible with the development of single-cell ATAC sequencing (scATAC-seq) strategies^7–9^. In ATAC-seq, Tn5 transposase, preloaded with next-generation sequencing adapters, simultaneously cuts and ligates sequencing adapters at accessible open chromatin regions^5,6^. The captured fragments between the adapters can then be amplified by PCR and subjected to high-throughput sequencing^5,6^.

Epigenetic mechanisms govern a significant aspect of gene expression machinery critical to cell development and differentiation^10,11^. Epigenetic perturbations and altered gene expression have been reported in many autoimmune disorders such as systemic lupus erythematosus (SLE), inflammatory bowel disease (IBD), rheumatoid arthritis (RA) and multiple sclerosis (MS)^12^. Profiling of chromatin accessibility in disease cohorts will increase our understanding of dysregulation of epigenetics in autoimmunity, and greatly aid the development of translational research and personalized medicine through identification of disease-relevant epigenetic signatures. Although the protocol is widely applied on freshly processed samples only a number of studies have examined the effect of cryopreservation on chromatin structure and gene expression^13–19^, none to date have been performed on primary human T cell subsets including cryopreserved human FOXP3^+^ Regulatory T (Treg) cells*.* FOXP3^+^ Regulatory T (Treg) cells are a rare subset of lymphocytes that maintain immune tolerance and prevent inappropriate immune responses^20–23^. Alterations in the frequency and function of Treg cells have been implicated in a wide range of autoimmune disorders including Type 1 diabetes (T1D)^24^, SLE^25^, IBD^26^ and RA^27^.

Cryopreservation of intact living cells and tissues is a common practice in basic and clinical research applications, allowing for the following of patients or samples across symptom states and over time. Cryopreservation becomes critical in large cohort studies where large sample numbers or many treatments or time points preclude genomics on fresh samples. Such biorepositories are increasingly making a significant contribution to improving our understanding, diagnosis, prevention and cure of complex diseases^28,29^. Significant improvements in the collection and storage of human biological samples have been observed in the past few decades^29^, allowing the identification of disease biomarkers to inform diagnosis and prognosis, as well as drug development. Biobanking is increasingly being employed in many fields including autoimmunity. In applying these cutting-edge genomic approaches to biobanked material, it is imperative therefore to ensure that recovered cells closely recapitulate the phenotype of the fresh cells for accurate interpretation and clinical translation, however, reports probing the effects of cryopreservation on the epigenetics of the recovered cells are limited^13,14^.

For high quality multi-omics analysis to be performed on these biobanks, not only is the post-thaw viability and recovery an important metric, but the recovered samples should closely reflect the physiological and biochemical state of their pre-freeze state. To test this, we adopted biobanking standards established by the Environmental Determinants of Islet Autoimmunity (ENDIA)^30,31^ study for cryopreservation of PBMCs obtained from healthy human adult subjects. Our study set out with the aim of assessing the global chromatin accessibility landscape of cryopreserved, rare immune cell populations, and tested the feasibility of quantifying chromatin accessibility and transcriptome in parallel from a single reaction of 50,000 cells. Specifically, we asked whether freezing before nuclei isolation compromises nuclear integrity and alters chromatin structure in FOXP3^+^ Regulatory T (Treg) cells.

As immune cells need to respond to environmental stimuli, they have highly co-ordinated transcriptional programs that drive the induction of immune response genes with specialized functions for an appropriate immune response against invading pathogens and cancer. Dysregulation of these transcriptional programs on the other hand is associated with autoimmunity. The sensitivity and responsiveness of cells to external stimuli critically dictate the magnitude of chromatin accessibility and gene expression changes, and disturbance of this may be the driver of disease. To apply this in an immune cell context, we adopted Omni ATAC-seq^32^ because it was shown to deliver substantial improvement in data quality across multiple applications, including frozen materials. ATAC-seq was performed on Treg cells isolated from peripheral blood mononuclear cells (PBMCs) obtained from healthy human adult subjects, processed fresh or cryogenically stored and thawed according to published biobanking protocols^31^. We profiled chromatin accessibility and TF occupancy in Treg cells in the steady state and in response to stimulation. We demonstrated that isolation of intact nuclei from both freshly processed and thawed samples produced chromatin accessibility patterns that were virtually indistinguishable between groups. In addition, we describe a workflow that incorporates ATAC-seq and RNA-seq to measure chromatin accessibility and gene expression simultaneously in 50,000 cells, allowing inference of regulatory association between the two systems from the same pool of cells. In the ATAC-seq workflow, following gentle cell lysis the nuclear and cytoplasmic fractions are separated through centrifugation. The nucleus is subjected to ATAC-seq whereas the supernatant fraction containing the cytoplasmic fraction is subjected to RNA-seq. We observed a high concordance in the transcriptomic profile between RNA purified from whole cells and ATAC-seq supernatant fractions from cryopreserved samples, indicating that parallel profiling of chromatin accessibility and gene expression is a valid approach in experiments constrained by input material. In summary, we demonstrate that a combined ATAC-seq and RNA-seq analysis can be applied to pooled biobanked cells, and the results show a strong correlation with the same assays performed on freshly isolated cells, whether resting or activated. This provides confidence when applying these approaches to biobanked immune cells from clinical cohorts.

## Results

### The complexity of T cell populations is preserved in cryopreserved PBMC samples

**Figure 1:**
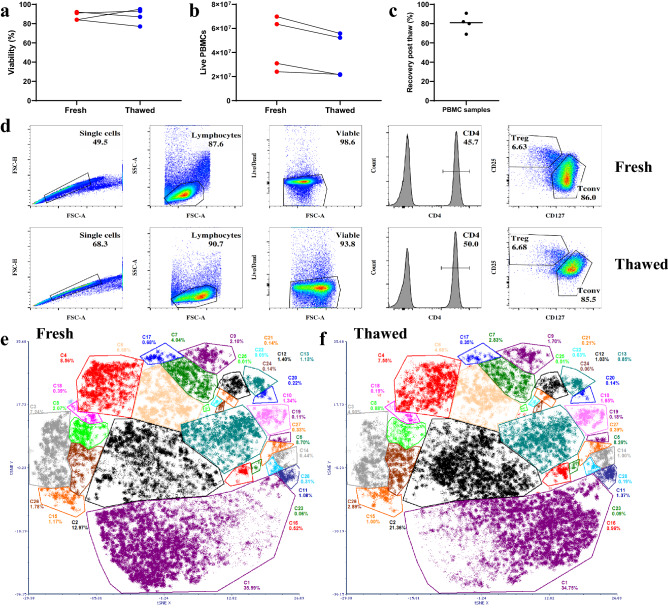
The complexity of frozen samples recapitulates that of the fresh samples. Viability (**a**) and absolute cell count (**b**) of the fresh and thawed PBMC samples. (**c**) Recovery (%) of live PBMC recovery after thawing. The cell viability and recovery of PBMC samples were determined using trypan blue dye exclusion test. (**d**) Flow cytometric profile from a representative fresh and thawed PBMCs sample and the gating strategy to isolate Conventional T (Tconv) and Regulatory T (Treg) cells. (**e**–**f**) Colour dot plots showing events from fresh (**e**) and thawed (**f**) CD4 positive cells contributing to t-SNE (t-distributed stochastic neighbour embedding) distribution with manual clustering.

To test whether frozen biobanked material faithfully reflects fresh material, each PBMC sample obtained from a healthy human adult subject was divided in half, one was processed immediately, and the other half cryopreserved and stored before thawing for cell isolation and ATAC-seq and RNA-seq experiments. Cryogenic storage did not significantly affect cell viability, recovery or frequency of CD4^+^ Tconv and Treg cells (Fig. 1a–d). Furthermore, the freezing process preserved T cell complexity, as demonstrated by the flow cytometry analysis (Fig. 1d) as well as preservation of T cell subsets identified through t-distributed stochastic neighbour embedding (t-SNE) analysis (Fig. 1e,f and Supplementary Fig. 1). Well-formed cell population clusters were identified in both fresh and thawed CD4^+^ T cells (Fig. 1c) and they include populations expressing T cell signatures such as FOXP3, CD25, CD127, CD45RA and CCR10 (Supplementary Fig. 1). All of these data demonstrate that T cells isolated from thawed material are viable and exbibit cell surface markers similar to that of the freshly processed cells.

### Chromatin accessibility signal is preserved through cryopreservation

**Figure 2:**
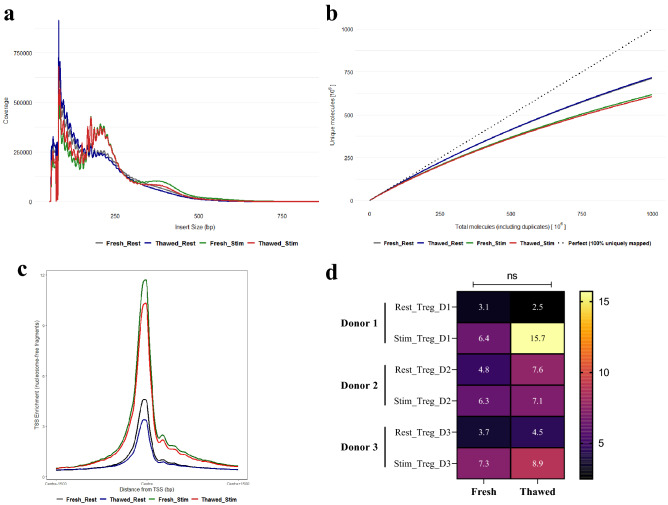
The quality of ATAC-seq from thawed Treg cells closely recapitulates that of the fresh cells. (**a**) The insert size distribution of fresh and thawed Treg ATAC-seq libraries. (**b**) Full library complexity and extrapolated yield curve of fresh and thawed ATAC-seq libraries. Complexity measurements are plotted against highest complexity (100% uniquely mapped reads). (**c**) Distribution of ATAC-seq signal at ± 1.5 kb transcriptional start sites (TSS). Signal coverage is calculated from reads per million mapped reads for each sample. (**d**) Percentage of reads mapping to mitochondrial genome. Deeper colour is used to depict the most desirable value of the statistic and range following a linear scale starting at 0 (black) and ending at the maximum value (yellow). All values were determined from the full depth of aligned reads. Statistical significance of the difference between the fresh and thawed samples is computed by Paired Student’s t-Test. The data shown in a-c are representative of sequencing reads pooled from three donors.

To ask whether the cryopreservation process alters the chromatin accessibility landscape of Regulatory T (Treg) cells, we performed Omni ATAC-seq on CD4^+^ CD25^hi^ CD127^lo^ Treg cells from PBMC obtained from healthy human adult subjects. PBMC samples were split and either processed immediately or cryogenically stored prior to CD4^+^ Treg isolation (Supplementary Fig. 2). We adopted Omni ATAC-seq as it was known to deliver high read mapping quality and library complexity^33^ and we confirmed this with our own analysis of peak identification on model datasets (Supplementary Fig. 11). To further probe the responsiveness of recovered Treg, isolated Treg cells were either left untreated (resting) or stimulated using Dynabeads Human T-Expander CD3/CD28 for 48 h prior to Omni ATAC-seq (Supplementary Table 1 for sequencing output). We identified an average of 41,587 reproducible peaks for fresh (resting) samples, 45,534 reproducible peaks for thawed (resting) samples, 88,631 reproducible peaks for fresh (stimulated) samples and 95,670 reproducible peaks for thawed (stimulated) samples (Supplementary Fig. 5a). There was no significant difference in the number of reproducible peaks between donor pairs in both fresh and cryopreserved groups (Supplementary Fig. 5b). The fragment size distribution of the amplified ATAC-seq libraries generated from fresh and thawed Treg cells showed a clear, characteristic nucleosomal laddering pattern with a periodicity of 200 bp, corresponding to DNA fragments from nucleosome-free regions (NFRs) or regions protected by integer multiples of phased nucleosomes (Fig. 2a, Supplementary Figs. 3 and 4), indicative of quality ATAC-seq libraries. The thawed Treg cells demonstrate a similar structured ATAC-seq signal around nucleosomes as the fresh samples (Supplementary Fig. 4) with both fresh and thawed Treg ATAC-seq profiles showing a distinctive V-shaped pattern where the apex represents the shortest possible fragment that is protected by a nucleosome (~ 117 bp). The most enriched position in the V-plot corresponds to fragments of 143 bp centered at the nucleosome dyad, which is the length of DNA bound by a canonical nucleosome. This pattern of chromatin architecture organization is in agreement with those obtained by Schep et al.^34^. A more striking nucleosomal periodicity pattern was observed upon stimulation in both fresh and thawed Treg cells compared with their resting counterparts (Supplementary Fig. 4). This suggests that stimulation results in significant alterations in nucleosome remodelling and chromatin accessibility. These results confirm that freezing did not disrupt the displacement and phasing of nucleosomes along the DNA sequence.

**Figure 3:**
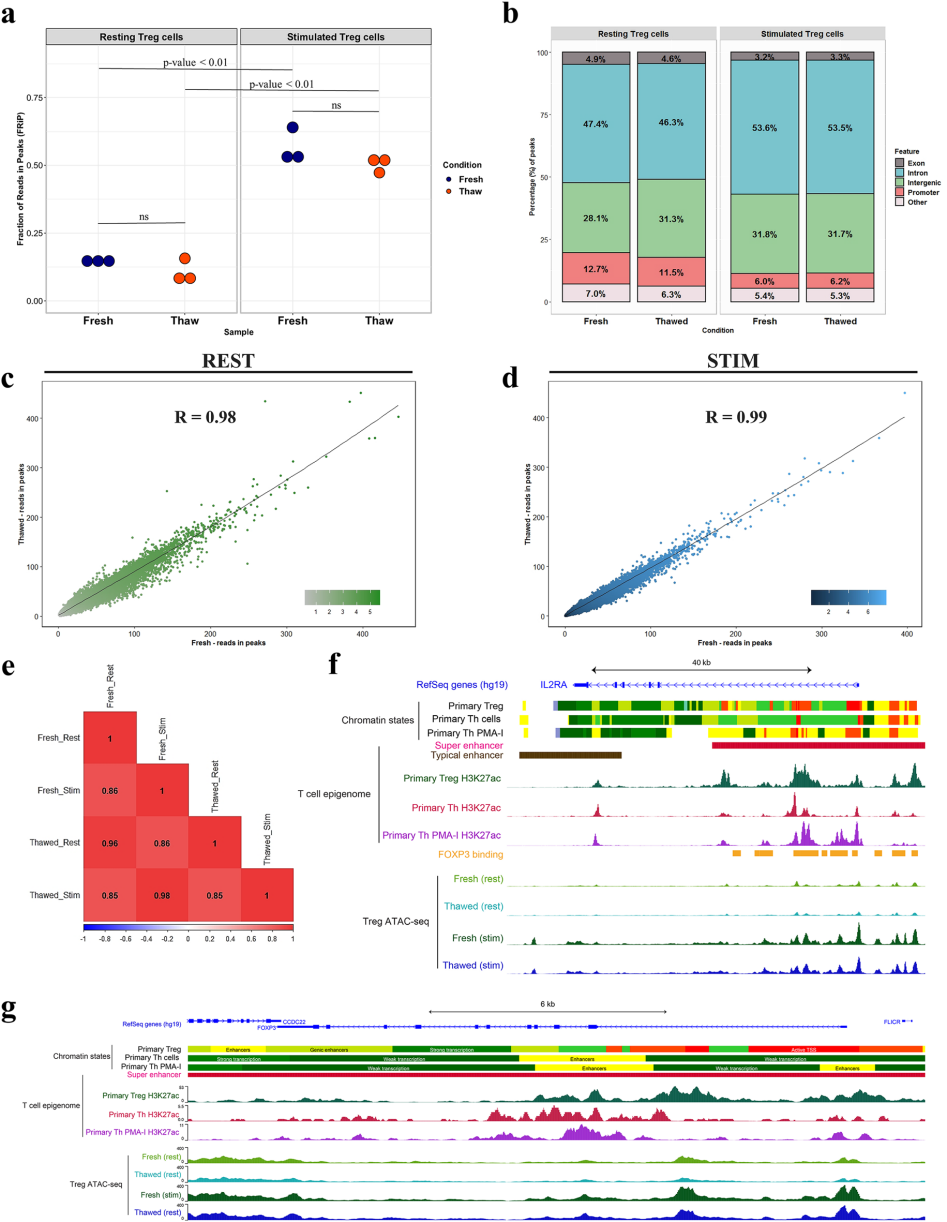
Chromatin accessibility is maintained through cryopreservation. (**a**) The fraction of reads in peaks for fresh and thawed Treg ATAC-seq libraries. Statistical significance of the difference between the fresh and thawed samples is computed by Paired Student’s t-Test. (**b**) Distribution of ATAC-seq peaks across distinct genomic feature expressed in proportion of annotated peaks. Promoters are defined by -1 kb to + 100 bp TSS region. Other, peaks annotated to 3’ UTR, 5’UTR, miRNA, non-coding RNA and TTS (transcription termination site). (**c**, **d**) Scatter plot of the read count per million (CPM) reads in ATAC-seq peaks identified from fresh and thawed samples during resting (**c**) and in response to stimulation (d). Pearson’s correlation coefficient value is indicated. (**e**) Correlogram showing the association of CPM reads for all the ATAC-seq samples generated for this study. (**f**, **g**) Chromatin accessibility profiles of fresh and thawed Treg cells during resting and stimulated state at the *IL2RA* (**f**) and *FOXP3* (**g**) loci. ATAC-seq signal is intersected with T cell super enhancers, Treg chromatin states from Roadmap Epigenomics Project and FOXP3 binding sites. Each ATAC-seq track represents signal pooled from three donors. Browser view was generated using UCSC genome browser. The calculation for a-g was performed on pooled data representative of three donors.

The fresh and thawed libraries demonstrated comparable molecular complexity (Fig. 2b) and enrichment of transcription start sites (TSSs) (Fig. 2c), a key measure of signal-to-noise ratio as recommended by ENCODE^4^, in both resting and stimulated states. No significant difference was observed in the proportion of mitochondrial reads between the ATAC-seq libraries generated from the fresh and thawed Treg cells (Fig. 2d). We also observed no significant difference in the fraction of mapped reads in the ATAC-seq peak regions (FRiP) (Fig. 3a) or their genomic distribution (Fig. 3b) in freshly processed compared with frozen samples, suggesting cryopreservation did not alter the transposition efficiency at accessible chromatin regions. An equivalent increase in the reads per peak following cell stimulation was indicative that cryopreservation did not grossly affect the changes in chromatin organisation upon cell activation (Fig. 3a and Supplementary Fig. 5a). Furthermore, the fresh and thawed samples showed a high correlation of peak signals indicating freeze-thaw did not have an appreciable impact on the accessible chromatin landscape in either resting or stimulated cells (Fig. 3c–e, Supplementary Fig. 6). The chromatin accessibility landscape at loci indispensable for Treg function and development such as *IL2RA* (Interleukin 2 Receptor Subunit Alpha) and *FOXP3* (Forkhead box P3) were also found to be highly preserved (Fig. 3f,g). Together these observations suggest cryopreservation does not perturb cellular responses, chromatin landscape and epigenetic signatures and the protocol can be applied on cryopreserved primary human Treg cells.

### Cryopreservation does not perturb cell responsiveness to stimulation and TF-DNA interaction

**Figure 4:**
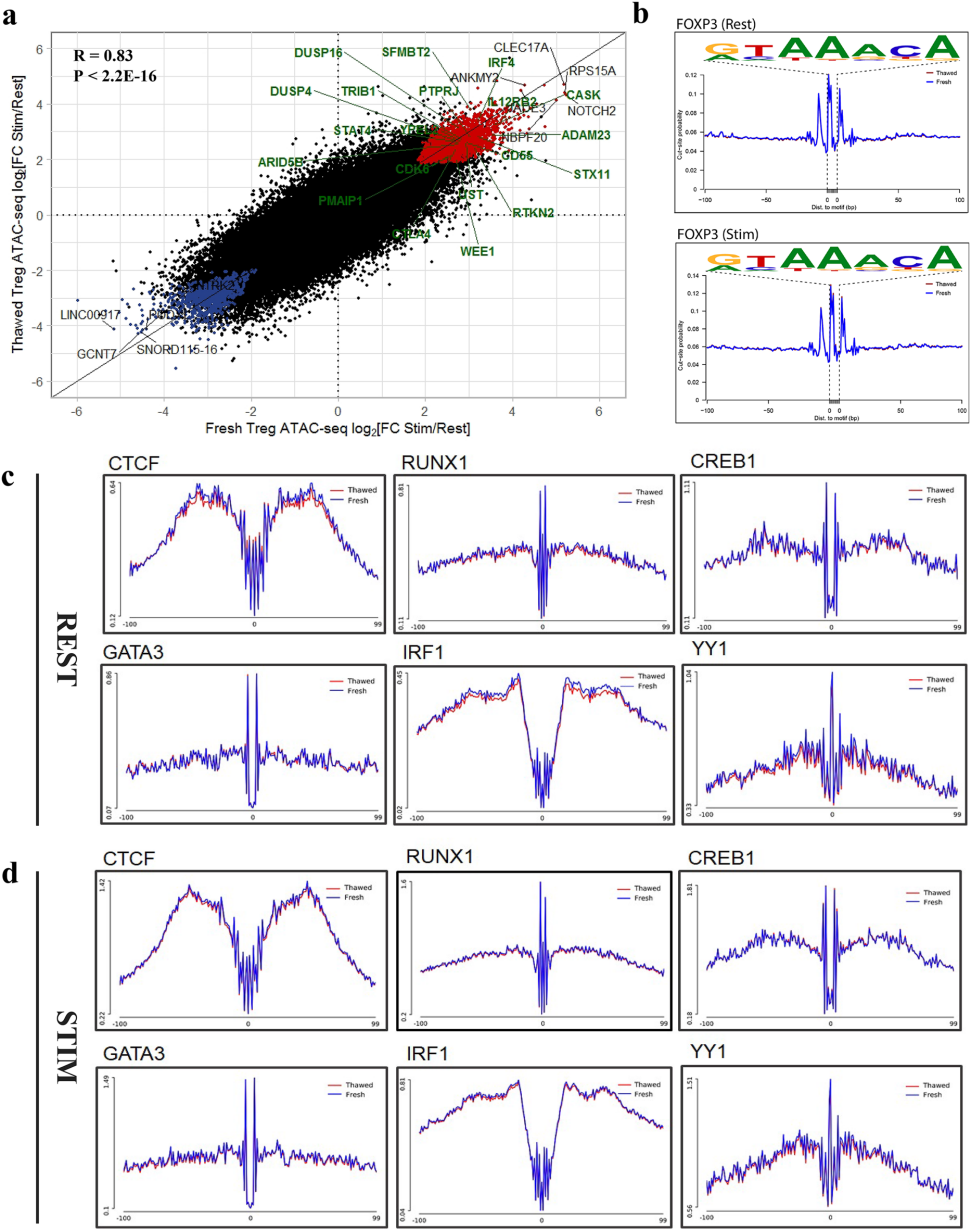
Thawed Treg cells demonstrate comparable TF activity to fresh cells. ATAC-seq was performed on sorted resting and stimulated fresh and thawed Treg cells. (**a**) Correlation of stimulation-responsive chromatin accessibility changes in fresh and thawed Treg cells (expressed as fold changes in reads in peaks between resting and stimulated states). Differentially accessible genomic loci between resting and stimulated states were defined as genes having an FDR of less than 0.05 and log-fold change that is significantly greater (red) or lower (blue) than 1.2 (equivalent to a 2.3-fold difference between conditions). ATAC-seq peaks were annotated to the nearest TSS and common differentially accessible peaks with a log-fold change that is significantly greater/lower than 4 are annotated by gene symbol. Top 20 common differentially accessible Treg signature genes (Ferraro et al. ^37^) are highlighted in green. (**b–d**) Histogram comparing fragments from fresh and thawed samples at FOXP3 (**b**), CTCF, RUNX1, CREB1, GATA3, IRF1 and YY1 binding motifs during resting (**c**) and stimulated (**d**) states. TF occupancy signal is computed on the genome-wide ATAC-seq footprints matching the corresponding motifs obtained from JASPAR database. Histograms are generated from Treg chromatin accessibility signals computed from pooled sequencing data representative of three donors.

**Figure 5:**
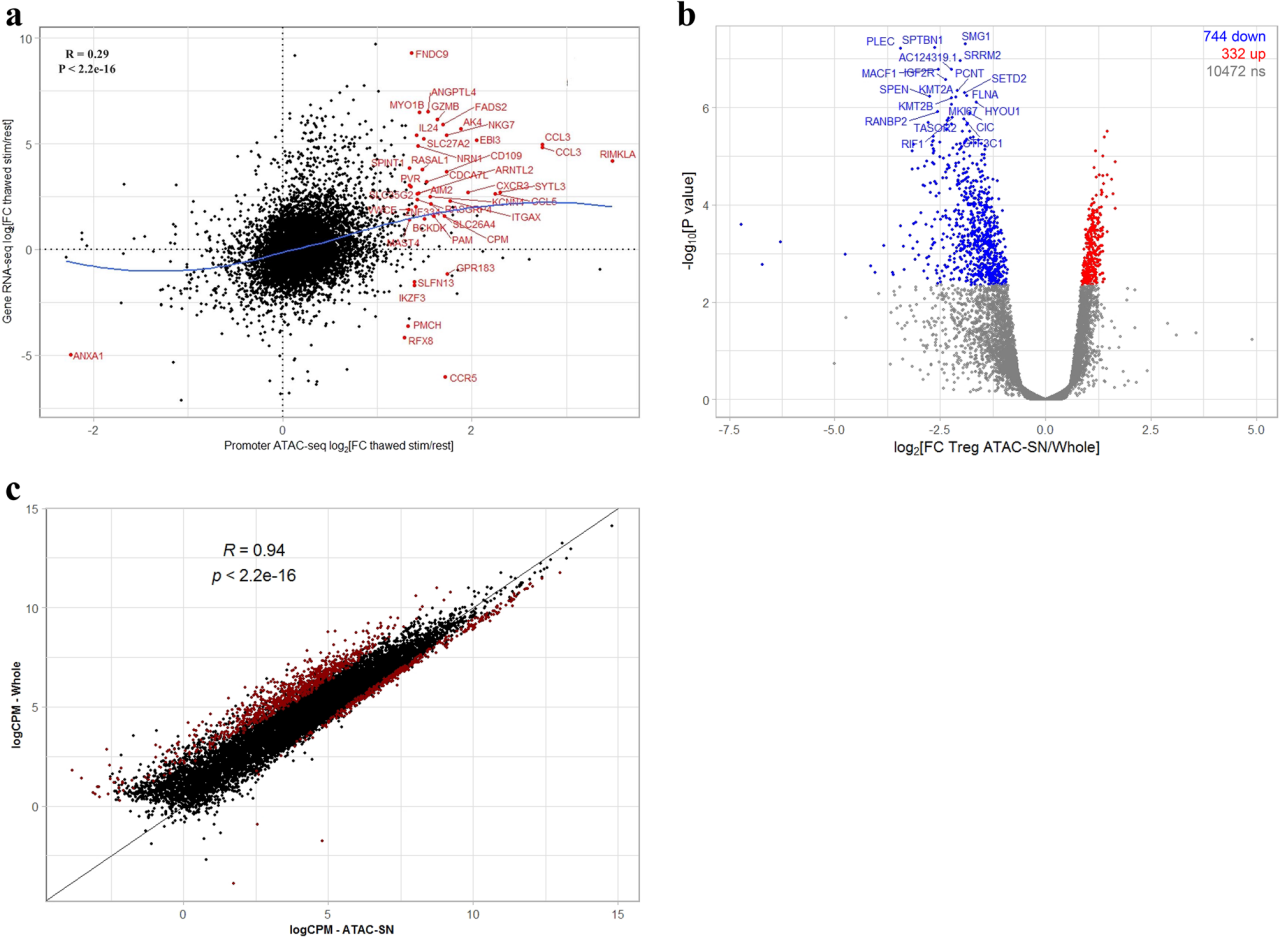
Parallel measurement of chromatin accessibility and transcriptome dynamics in human Treg cells. RNA-seq was performed using RNA isolated from whole cells or supernatant (cytoplasmic) fraction recovered from ATAC-seq reaction prepared from thawed Treg cells. (**a**) Correlation of stimulation-responsive chromatin accessibility and gene expression changes in thawed, whole Treg cells (expressed as fold changes between resting and stimulated states). Differentially accessible promoter/expressed genes between resting and stimulated states were defined as genes having an FDR of less than 0.05 and log-fold change that is significantly greater or lower than 1.5. Promoters having differential chromatin accessibility and expression changes upon stimulation are highlighted. Blue line indicates loess fit to the distribution. (**b**) Differential analysis of transcriptomic profiles generated from ATAC-seq supernatant (cytoplasmic) fraction and whole Treg cells. Differentially expressed genes were defined as genes having an FDR of less than 0.05 and log-fold change that is significantly greater (red) or lower (blue) than 1.5. Top 20 differentially accessible genes are annotated by gene symbols. (**c**) Correlation of transcriptomic profiles generated from ATAC-seq supernatant fraction and whole cells. The expression for each gene is represented by log count per million (CPM) of sequencing reads. Differentially expressed genes are highlighted in red. Results shown are representative of four donors.

Immune cells respond to environmental stimuli constantly with reprogramming of chromatin domains, resulting in transcriptional programs that drive the induction of immune response genes with specialized functions. T-cell stimulation-specific genomic regions are enriched for GWAS-eQTLs or SNPs associated with autoimmune diseases such as IBD and RA, as well as T cell subset specific enhancers, emphasising the importance of profiling immune cells under stimulation to identify disease-relevant regulatory elements and mechanisms^35,36^. In accordance with previous studies^35,36^, stimulation results in a global increase in chromatin changes. High concordance of genome-wide chromatin accessibility changes and TF occupancy were observed in the fresh and thawed Treg cells in response to stimulation (Fig. 4a, Supplementary Fig. 7 and Supplementary Table 2), including at classic Treg signature genes^37^. We extended this analysis to assess the correlation between promoter accessibility signal and corresponding gene expression generated from thawed Treg cells using whole cell RNA-seq, as the general consensus is that an accessible promoter is indicative of active gene expression. In agreement with other studies^38,39^, the correlation between promoter accessibility and respective gene expression was significant (Fig. 5a and Supplementary Table 2), suggesting freezing did not impair the induction of activation-responsive epigenome and gene expression in Treg cells.

### Transcription factor activity is conserved through cryopreservation

Within accessible chromatin, transcription factor (TF)-bound DNA sequences are selectively resistant to Tn5 cleavage, resulting in short regions of protected DNA or footprints. Footprint analysis of Omni ATAC-seq was carried out with pooled accessible fragments representing nucleosome-free regions (NFRs) and regions bound by one nucleosome (1N)^40^. A total of 551 TF footprints were discovered in our Treg ATAC-seq data (Supplementary Table 3). We observed clear, discrete TF footprints in accessible regions identified from our fresh and thawed Treg cells during resting state and in response to stimulation (Fig. 4b–d). Overlay of the genome wide footprint signal associated with fresh and thawed Treg samples indicated similar accessibility levels were observed surrounding the positions of predicted TF binding sites for a wide range of TFs, including FOXP3, which is the master TF of Treg cells (Fig. 4b), as well as TF with important roles in T cells such as CTCF, RUNX1, CREB1, GATA3, IRF1 and YY1 (Fig. 4c,d). These data strongly indicate an indistinguishable chromatin landscape and TF occupancy in open chromatin regions in fresh and thawed Treg cells, and freezing or biobanking did not affect the ability to resolve the accessibility patterns of single base-pair TF occupancy and had no global effect on TF-DNA interactions.

### Simultaneous profiling of chromatin accessibility and transcriptome in cryopreserved primary Treg cells

Parallel profiling of chromatin accessibility and the transcriptome within the same pool of cells allows for the unravelling of the complex relationship between regulatory elements and gene expression programs. This approach will be complementary to single-cell experiments as libraries used to profile chromatin accessibility and transcriptome are derived from the same population of cells, controlling for stochastic gene fluctuation in different cells in a population at any given time. This workflow is particularly beneficial for experiments constrained by limited input material such as the use of cryopreserved, rare human cell population. In the ATAC-seq workflow, the whole cell lysates are separated into nuclear and cytoplasmic fractions through centrifugation. We subjected the nucleus to ATAC-seq, whereas RNA was isolated from the supernatant component and subjected to RNA-seq. In evaluating the feasibility of using RNA recovered from cytoplasm-derived ATAC-seq supernatant (ATAC-SN), we first assessed the global gene expression patterns of ATAC-SN compared to total RNA isolated from intact human Treg cells. The ATAC-SN and whole cell fraction exhibited a significantly high correlation (R = 0.94; *p*-value < 2.2 × 10^–16^) of transcript abundance, where 90.7% (10,472 out of 11,548 genes) of the transcriptome was not significantly altered in both compartments (Fig. 5b,c and Supplementary Table 2). The criterion for significance was defined as a false discovery rate (FDR) of less than 0.05. These confirm that there is minimal bias introduced by the method.

A small fraction (9.4%) of genes were differentially enriched in the cytoplasmic-derived ATAC-SN vs. the whole cell compartments. The majority of the genes accounting for differences in expression levels were genes downregulated in the ATAC-SN fraction. Gene ontology (GO) enrichment analyses were performed for genes that were significantly more abundant or depleted in the ATAC-SN fraction. Transcripts that were under-represented in the ATAC-SN were associated with nuclear biological processes and molecular functions, including nuclear division, histone modification, nuclear division, chromosome organization and glycoprotein (Supplementary Fig. 8a, c). Furthermore, our results also showed that genes that were detected at lower levels in the ATAC-SN with respect to the whole cell fraction were associated with reduced RNA stability in the human HapMap lymphoblastoid cell lines (LCLs),^41^ (Supplementary Fig. 9). In contrast, transcripts that were enriched in the ATAC-SN fraction were involved in membrane-associated processes such as co-translational protein targeting to membrane, protein targeting to endoplasmic reticulum (ER), as well as cytoplasmic translation, mitochondrion organization, and ribosome biogenesis (Supplementary Fig. 8b,d). These results show high overlap with studies in other tissues that identified differential enrichment of mRNA molecules in the nuclear and cytoplasmic compartments^42–44^. For instance, it was demonstrated in Zaghlool et al.^44^ that many nuclear-encoded-mitochondrial protein (NEMPs) mRNAs are significantly enriched in the cytosolic compartment across various human tissue types. To determine if NEMPs mRNAs were also preferentially localized in the ATAC-SN fraction of human T cells, we retrieved a full list of genes (n = 1158) encoding proteins associated with mitochondrial localization from Mitocharta2.0^45^ and conducted gene enrichment analysis for differentially expressed genes either up- or down-regulated in the ATAC-SN fraction and human NEMPs. In agreement with the previous study, we observed an abundance of NEMP transcripts in the cytosolic compartment. Our results show that a total of 71 (20.6%) genes significantly upregulated in the cytoplasmic-derived ATAC-SN overlapped with the human NEMPs dataset, whereas only 13 (1.6%) genes downregulated in the ATAC-SN were NEMPs (Supplementary Fig. 10a). NEMPs were shown to be significantly enriched only in the ATAC-SN fraction (p-value = 6 × 10^–10^) and not in whole cellular extracts (Supplementary Fig. 10b), consistent with the ATAC-SN fraction being primarily cytosolic-derived. Overall, we conclude that the transcriptomes derived from the ATAC-SN and whole cells exhibited a high degree of concordance (> 90%) and that ATAC-SN can be used to study global gene expression patterns from low input resources such as rare cell populations or clinical samples. Our findings also indicate that ATAC-SN preserves subcellular localization of cytoplasmic enriched mRNA, as confirmed by enrichment of NEMPs.

## Discussion

Although it has been postulated that cryopreservation can impact the viability and functionality of PBMCs^46,47^, and repeated freeze-thaw cycles have a negative impact on cell viability and function^48^, the use of fresh samples from large cohorts or longitudinal studies poses several batch effect challenges, and bio-banking mitigates these. The potential for introducing confounding variables in downstream assays is higher for fresh samples processed over a prolonged length of time compared with batched cryopreserved samples. In addition, sole reliance on fresh samples prevents access by future research for validation studies. ATAC-seq is widely applied on freshly processed samples and only a limited number of studies have been performed to benchmark the effects of cryopreservation on chromatin structure^13,14^. In this study, we demonstrated that ATAC-seq can be reliably used to assess the global chromatin accessibility landscape of primary regulatory T cells (Treg) recovered from cryopreserved human peripheral blood, and this is likely amenable to other T cell or immune subtypes. We first demonstrated that cryopreservation had no significant effect on T cell viability or population complexity. Using criteria recommended by the ENCODE consortium^4^ we demonstrated that ATAC-seq libraries prepared from thawed cells delivered high signal-to-noise ratio, comparable with the libraries prepared from freshly processed cells. We further observed high quality chromatin accessibility data in cryopreserved Treg cells with respect to library complexity, TF occupancy and cell responsiveness to stimulation, which was highly similar to the results obtained with freshly isolated cells. Lastly the results of this study show that the accessibility signal at gene loci and TF occupancy footprints essential for Treg activation and function were well conserved in cells recovered from cryopreservation, reinforcing that the process did not disrupt the epigenetic signatures critical for the regulation of transcriptome in Treg cells. These findings reflect those of Scharer et al.^13^ and Fujiwara et al.^14^ in primary B cells and breast cancer cell lines, who also found high correlation of signal to noise ratios, accessibility levels and TF footprint patterns between fresh and biobanked specimens. High correlation between fresh and frozen cells has also been observed in single-cell ATAC-seq profiles generated from PBMC samples^49^.

While previous research has focused on the impact of freezing on the genome-wide chromatin architecture in cells in the steady state, our results build on this by demonstrating indistinguishable chromatin accessibility and TF occupancy in T cells isolated from biobanked PBMC samples under resting state and, critically in response to cell stimulation. Stimulation has been shown to result in global, large-scale changes in chromatin accessibility^35,36^. Dysregulation of immune homeostasis and activation are known to play a role in cancer and autoimmunity and hundreds of genetic variants have been associated with stimulation-specific transcriptional regulation and gene expression in T cells^50,51^. It is worth noting that, on the level of genome-wide chromatin accessibility, the differences captured between the fresh and thawed Treg ATAC-seq libraries were minimal and more appreciable changes were detected when comparing resting with stimulation in both the fresh and thawed samples (Supplementary Figs. 5 and 6). It is encouraging that the results of this study also indicate that the responsiveness to activation of Treg samples recovered from cryopreserved PBMC was almost indistinguishable from the fresh samples, confirming the biobanking process did not compromise the sensitivity of cells to respond to activation and epigenetic reprogramming of immune responses. Importantly, the chromatin accessibility of the cryopreserved samples was positively correlated with the expression of the target genes. This provides valuable insight into regulatory networks, which is not possible with just transcriptomic profiles alone. Taken together, this confirms that the biobanked samples can be reliably used to identify genetic or epigenetic drivers to reveal disease mechanisms resulting from immune dysregulation.

To extend the applicability of genomics to low cell number biobanked samples, or to rare cells in the blood, we also developed a workflow designed for parallel profiling of chromatin accessibility and transcriptome within the same population of 50,000 cells. This workflow was designed to enable experiments constrained by limited input material, such as the use of clinical paediatric samples or rare cell types. In the disease context, not only does bridging ATAC-seq and RNA-seq help us understand how epigenetics is altered, but it provides insight into the molecular mechanisms by which regulatory elements contribute to disease pathology. In the ATAC-seq protocol, upon cell lysis, centrifugation separates the lysed cellular material into two fractions—1) pellet, which contains intact nuclei, which is used for Tn5 transposition in ATAC-seq, and 2) supernatant, which contains lysates derived mainly from the cytoplasm and non-nuclear organelles. We established a protocol to profile the transcriptome from the nuclear lysate supernatant (‘‘ATAC-SN’’) and revealed a high correlation in the relative distribution of transcripts derived from ATAC-SN and total cellular extracts in primary human Treg cells. These results confirm that the majority of the transcriptome was congruent in both compartments, and a high proportion of genes were detected in similar amounts. Although transcriptome analysis such as RNA-seq and microarray has predominantly been performed using RNA extracted from whole cells, growing evidence is emphasising the importance of investigating the subcellular repertoire of RNA molecules and understanding the spatial dimension that governs their distribution inside the cell^52–55^. A majority of the ATAC-SN fraction comprises cytoplasmic RNA and studies have demonstrated that the cytoplasmic transcriptome strongly correlates with the whole cell transcriptome^42,56^. Cytoplasmic RNA may a better proxy for protein levels since the cytoplasmic fraction has been suggested to provide a more legitimate representation of steady-state mRNA^43,57^. Trask et al.^43^ described that the nuclear components must be excluded, or at least analysed separately from the cytoplasmic fractions for accurate and reproducible quantification of gene expression. It was reported that inclusion of nuclear RNA, which represents 10–15% of the total RNA^42^, distorts the mRNA profile and contributes to substantial level of false positives. That study argued that by omitting a subset of nuclear transcripts with variable or stochastic expression, differentially expressed genes that would have been missed or, statistically non-significant using the whole cell fractions, were recovered.

Overall, we detected a high overlap of transcriptomes (90.7%; R = 0.94) within comparisons of whole cell and ATAC-SN fractions (FDR < 0.05). Although it represents a small proportion of the total transcriptome, we did investigate the drivers of that difference. There was overall a higher number of downregulated genes in the ATAC-SN where the nucleus is absent, in comparison with the whole cell fraction. A small number of transcripts that were depleted in the ATAC-SN show an overrepresentation of biological processes and molecular functions associated with the nucleus, including nuclear division, nuclear membrane, histone modification, chromosome organization and glycoprotein. While this is unsurprising, as that compartment is technically excluded, so will appear as DE, there are processes linked to these transcripts. The rate of transportation from nucleus to cytoplasm can have an impact on the level of transcripts detected in both compartments, and it was reported that mRNA molecules that are not of immediate need for producing proteins are retained in the nucleus^57,58^. Transcription in mammals has been demonstrated to be a pulsatile process involving stochastic bursts of mRNA production followed by intervals of promoter quiescence^59^ and mRNA compartmentation acts to buffer these transcriptional fluctuations in the cytoplasm and eventually protein levels^58^. Nuclear retention of incompletely spliced^60^, mature or hyper-edited mRNAs^61^ also plays an important role in gene regulation, allowing the cell to rapidly respond to stress, viral infection, or changing environmental stimuli^62,63^. The proposed model for nuclear retention could explain the modest depletion of transcripts in the ATAC-SN fraction. In addition, transcripts with an intrinsically high rate of degradation in the cytoplasm may be detected at lower amounts in the cytoplasm than the whole cell fractions. Likewise, the enrichment of transcripts in the ATAC-SN with respect to the whole cell fraction could be attributed to their high stability in the cytoplasm and low transcription rates. Conversely, consistent with Zaghlool et al.^44^, the cytosol-derived ATAC-SN fraction showed enrichment of nuclear-encoded mitochondrial proteins (NEMPs). NEMPs mRNAs were known to accumulate in the cytoplasm until their translation is required by the mitochondria^64^ and have a significantly longer mRNA half-life compared with other protein-coding transcripts, which supports their preferential and prolonged localization in the ATAC-SN. Nonetheless, our data strongly suggest majority of the transcripts exhibit equal representation of abundance in the two compartments. Our results show that at a global level the transcriptome derived from ATAC-SN closely recapitulated that of the whole cell lysate, demonstrating the feasibility and reliability of using ATAC-SN and RNAseq combined workflow for a high resolution representation of gene regulation and expression levels in biobanked cells. This indicates that for most transcripts neither selective cellular localization nor RNA stability impairs their distribution within the cell.

While we recognise modest sample size can be a potential limitation of this study, we demonstrate high feasibility and reliability of profiling chromatin accessibility using ATAC-seq on human primary cells recovered from biobanked materials. These experiments were performed using 50,000 cells but in our other studies we have used as low as 25,000 cells and achieved similar results (data not shown). A further limitation is that we were not able to also examine the reproducibility of these omics approaches on normal tissue samples due to the ethical implications of sampling from healthy individuals, and perhaps tumour biopsies would provide and alternate resource to test this. However, our study is of significance as it shows that cryopreservation does not impair cellular responses, chromatin landscape and epigenetic signatures (gene accessibility signal and TF occupancy) of cryopreserved primary Treg cells, and the protocol can be used by research groups working with similar cell types of interest.

In conclusion, these findings have significant implications for studies that aim to obtain chromatin accessibility and transcriptome simultaneously from resources with limited input material, and until the depth, resolution and costs associated with the single cell technologies are the same as bulk approaches, there will still be a need for workflows that can use biobanked material and perform parallel discovery. Applying these techniques to biobanked material from disease cohorts knowing that the data from frozen material accurately reflects freshly isolated cells gives confidence that finding will provide valuable mechanistic insight into disease molecular profiles, opening the door to new therapeutic targets and personalized medicine.

## Methods

### Biobanking

Whole blood samples were collected with informed consent from four male healthy adult donors between 36 and 57 years of age. Samples were non-identifiable and no personal information was collected as part of the study. This research was approved by the Women’s and Children’s Health Network-Human Research Ethics Committee (WCHN-HREC) (approval code: HREC/19/WCHN/65; approval date: 11 June 2019) and all methods were performed in accordance with the relevant guidelines and regulations. PBMCs were isolated from fresh whole blood obtained from healthy adult donors by density gradient centrifugation using Ficoll-Paque solution. Briefly, blood samples collected in heparin-containing tubes were diluted at 1 × with Phosphate Buffered Saline and 35 mL of the diluted blood sample layered on 15 mL of Ficoll-Paque™ PLUS in 50-mL Falcon tubes, followed by centrifugation at 800 xg for 20 min in a swinging-bucket rotor with brake off. The mononuclear cell layer at the interphase was transferred to a 50 mL conical tube and topped up to a final volume of 50mLwith PBS. PBMCs were washed twice by centrifugation at 500 xg at RT for 10 min with the brake on. PBMCs were divided into two groups, half for fresh processing and the other half for freezing/biobanking. Samples to be frozen were resuspended in 1 mL of freezing medium (heat-inactivated FCS containing 10% DMSO) at a concentration of 1 × 10^7^ cells/mL, transferred to a 2-mL cryovial and placed into a Mr. Frosty™ Freezing Container for gradual freezing to − 80 °C at a rate -1 °C/minute. The cryovials were then moved to liquid nitrogen for long-term storage.

### PBMC thawing

Cryopreserved PBMCs were removed from liquid nitrogen and thawed in a 37 °C water bath for 10 min. Thawed PBMCs were transferred to a 10 mL conical tube and diluted with pre-warmed complete X-VIVO 15 culture media (X-VIVO 15 Serum-free media supplemented with 2 mM HEPES pH 7.8, 2 mM L-glutamine and 5% heat-inactivated human serum) at a rate of 1 mL/5 s. The 10 mL conical tube containing the cell suspension was gently inverted to mix and spun at 500 xg for 10 min at RT for a total of two washes. The cells were then resuspended in pre-warmed complete X-VIVO 15 culture media at 3–4 × 10^6^/mL. Upon thawing the PBMCs were allowed to recover overnight in complete X-VIVO 15 culture media in a 24-well culture plate prior to FACS.

### PBMC staining and FACS sorting of Regulatory T (Treg) cells

Following overnight resting in culture, freshly isolated or thawed PBMCs were washed in PBS supplemented with 2% FCS at 500 xg at RT for 10 min. Cells were labelled with the following fluorochrome-conjugated anti-human monoclonal antibodies: anti-CD4 (BD Biosciences, BUV395 Mouse Anti-Human), anti-CD25 (BD Biosciences, BV421), anti-CD127 (BD Biosciences, PE-CF594) and viability dye (BD Biosciences, BD Horizon Fixable Viability Stain 700) for FACS analysis. Treg cells were isolated as a CD4^+^ CD25^hi^ CD127^dim^ population using a BD FACSAria Fusion flow cytometer. A fraction of the isolated populations were analysed for cell purity by flow cytometry. Following cell sorting Treg cells were plated at 100,000 cells per well in a 96-well U-bottom plate and maintained in complete X-VIVO 15 culture media (X-VIVO 15 media supplemented with 2 mM HEPES pH 7.8, 2 mM L-glutamine and 5% heat-inactivated human serum) in the presence of 500U/mL recombinant human IL-2 for 2 h at 37 °C in a humidified 5% CO_2_ incubator prior to cell preparation for ATAC-seq and RNA-seq experiments.

### Stimulation of Regulatory T (Treg) cells

Following a 2-h recovery post sorting, Treg cells were either left untreated or stimulated with beads conjugated with anti-CD3 and anti-CD28 antibodies (Dynabeads Human T-Expander CD3/CD28, Gibco no. 11141D, Life Technologies) in complete X-VIVO 15 culture in the presence of 500U/mL recombinant human IL-2 at a cell/bead ratio of 1:1 for 48 h. After 48 h Dynabeads were removed from culture medium by magnetic separation.

### ***t-distributed stochastic neighbour embedding (t-SNE) analysis of Fresh vs Frozen CD4***^+^***T cells***

Fresh and thawed samples were stained with the following fluorescent conjugated antibodies (CD25-BV421, CXCR3-BV650, CCR6-BV786, CCR10-BB515, CD127-PE-CF594, CCR4-PE-Cy7, FOXP3-AF647 (PCT), CD45RA-APC-H7) and the LIVE/DEAD discrimination dye dye-AF700. Viable CD4^+^ T cell events (280,000) from fresh (n = 4) and thawed samples (n = 4) were subsequently exported for t-SNE analysis. The markers utilised are a defined phenotypic panel designed to determine distinct subpopulations in both Conventional T (Tconv) and Regulatory T (Treg) cells using chemokine receptors. Treg cells were defined from expression of CD25^+^, CD127^lo^ and FOXP3^+^. CD45RA is used to identify naive T cells (CD45RA^+^) and memory T cells (CD45RA^-^). The chemokine receptors CXCR3, CCR4, CCR6 and CCR10 are included as they are known to be expressed on helper lineage subpopulations Th1, Th2, Th17 and Th22 and can be found on Treg cells in a similar pattern. FOXP3 expression was transformed from mean fluorescence intensity (MFI) to percentage of Maximum MFI (designated ‘‘PCT’’) of each file prior to file concatenation. The MFI of CD25, CXCR3, CCR6, CCR10, CD127, CCR4, FOXP3(PCT), CD45RA were used in t-SNE machine learning on the total (1.12 × 10^6^) CD4^+^ T cell event dataset. The dataset was interval- down- sampled to 100,000 events before being subjected to 700 iterations of the tSNE equation with the following settings perplexity of 50 and a Barnes-Hut approximation of 0.5. Unsampled events resulting from down-sampling were estimated and plotted with the 100,000 events (FCS express v6). This allows mapping of unsampled events to their nearest sampled event for participation in the t-SNE calculation. Cell population clusters were determined from distribution and combinations of fluorescence markers.

### Omni ATAC-seq

Omni ATAC-seq was performed as described previously with minor modifications^32^. Briefly, cells were pretreated with 200U/µL DNase (Worthington) for 30 min at 37 °C prior to ATAC-seq experiment. Cell counts were performed manually using a hemocytometer and 50,000 Treg cells were lysed in 50µL of cold resuspension buffer (RSB: 10 mM Tris–HCl pH 7.4, 10 mM NaCl, and 3 mM MgCl_2_) supplemented with 0.1% NP40, 0.1% Tween-20, and 0.01% digitonin on ice for 3 min. Immediately after lysis, the reaction was washed with 1 mL of ATAC-seq RSB containing 0.1% Tween-20 by centrifugation at 500 xg for 10 min at 4 °C. After pelleting of the nuclei, the supernatant was collected and 3 volumes of TRIzol LS reagent (Invitrogen, 10296010) were added to the supernatant (divided into five 1.5 mL Eppendorf tubes) and stored at − 80 °C for RNA-seq experiments. The nuclei were resuspended in 50µL of transposition mix (30µL 2 × TD buffer, 3.0µL Tn5 transposase, 16.5µL PBS, 0.5µL 1% digitonin and 0.5µL 10% Tween-20). TD buffer and Tn5 transposase were purchased from Illumina Inc. The transposition reaction was incubated at 37 °C for 45 min in a thermomixer with 1000 rpm mixing. The reaction was purified using a Zymo DNA Clean & Concentrator-5 (D4014) kit. The subsequent ATAC-seq library preparation was performed as described^32^. All libraries were amplified for a total of 9 PCR cycles and size selection was carried out using SPRIselect (Beckman Coulter) to include a fragment size window of 100 to 800 bp prior to sequencing. Barcoded libraries were pooled and sequenced on a paired-end 75-cycle Illumina NextSeq 550 High-Output platform to an average read depth of 37.2 million reads (± 6 million) per sample (Supplementary Table 1).

### RNA isolation and library preparation for RNA-seq

RNA-seq profiles were collected from 50,000 whole cells (Resting and stimulated Treg cells) and supernatant fractions (stimulated Treg cells only) from Omni ATAC-seq lysis reactions (ATAC-SN) for 4 individuals, of which 3 have matching ATAC-seq profiles. Samples were homogenized using TRIzol LS reagent (Invitrogen, 10296010) and total RNA was extracted using a miRNeasy Micro Kit (Qiagen; cat# 217084). RNA integrity was determined by the Agilent RNA 6000 Pico Kit using an Agilent Bioanalyzer. Samples with RNA integrity number (RIN) > 7.5 were used for mRNA sequencing. The RNA samples were enriched for Poly(A) RNA using the NEBNext Poly(A) mRNA Magnetic Isolation Module (New England Biolabs #E7490) prior to generation of cDNA libraries using a NEBNext Ultra Directional II RNA Library Preparation Kit for Illumina (New England Biolabs #E7760S) according to the manufacturer’s protocol. Barcoded libraries were pooled and sequenced on a paired-end 150-cycle Illumina Hiseq X sequencer to an average read depth of 37.5 million reads (± 16 million) per sample.

### ATAC-seq data processing

ATAC-seq data analysis used the following tools and versions: FastQC ver. 0.11.7, Samtools ver. 1.3.1, Picard ver. 2.2.4, Bowtie2 ver. 2.2.9, MACS2 ver. 2.1.2, BEDTools ver. 2.25.0, bedmap ver 2.4.36, Subread ver. 1.5.2.

The sequencing data quality was determined using FastQC (ver. 0.11.7) followed by trimming of Nextera adapters using cutadapt (ver. 1.14). Trimmed reads were aligned to the GRCh37 genome using Bowtie2 (ver. 2.2.9) with ‘-X 2000’ setting. For each sample quality trimming was performed with option ‘-q 10’ with unmapped and non-primary mapped reads filtered with option ‘-F 2828’ using Samtools ver. 1.3.1. Uniquely mapped paired reads were then filtered to exclude PCR duplicates using Picard ver. 2.2.4. Mitochondrial reads, reads mapping to ENCODE hg19 blacklisted regions (regions with anomalous high signal across multiple genomic assays and cell types) and mitochondrial blacklisted regions (high signal regions on the nuclear genome due to sequence homology with the mitochondrial genome) were filtered out using BEDTools ver. 2.25.0. After filtering, a median of 28 million reads (± 3 million) reads per sample were obtained. For peak calling and TF footprinting, the read start sites were adjusted to represent the center of Tn5 transposase binding event. As Tn5 transposase binds as a dimer and inserts two adapters separated by 9 bp^65^ thus, all reads aligning to the forward strand were offset by + 4 bp whereas all reads aligning to the reverse strand were offset by − 5 bp.

### Differential ATAC-seq analysis

For differential accessibility between the resting and stimulated Treg samples, the processed ATAC-seq reads representing fresh and frozen samples from either resting or stimulated were concatenated. Peaks were called from the merged bam files using MACS2 ver. 2.1.2^66^ with parameters ‘callpeak -f BAMPE -g hs -nolambda -min-length 100 -max-gap 50 -call-summits -bdg -keep-dup all -p 0.1’. For each peakset overlapping peaks were merged using BEDTools ver. 2.25.0 and the number of reads in each individual sample/donor mapping to each peak was calculated using featureCounts^67^, considering only fragments with both ends successfully aligned (-B) and with reads overlapping multiple features assigned to the feature with the largest overlap (-largestOverlap). Count data were then imported into R and processed using edgeR^68^, removing any peaks having less than 3 counts per million in more than three samples. Counts were normalized for sample library size and common, trended, and tagwise dispersions were calculated. Differential accessibility analysis was performed using voom from limma package^69^. Significant differential accessibility was defined as regions having a Benjamini–Hochberg FDR below 0.05 and fold change greater than 1.5. IGV ver. 2.5.2 (Broad Institute) and UCSC genome browser (University of California Santa Cruz) were used for visualization of ATAC- and RNA-seq tracks. Peaks were annotated to the nearest TSS using ChIPpeakAnno^70^. HINT-ATAC^40^ was used to call footprints under the ATAC-seq peaks from the ATAC-seq data with parameters ‘-atac-seq -paired-end -organism = hg19’.

### Differential RNA-seq analysis

All RNA-seq samples were first validated for consistent quality using FastQC v0.11.7 (Babraham Institute) using default parameters. Raw reads were trimmed to remove adapters and low quality bases using AdapterRemoval/2.2.1 with parameters ‘-minquality 30 -minlength 50’. Adapter- and quality-trimmed reads were subsequently pseudo-aligned to the GRCh38 human genome using Kallisto with parameters’ -b 50 and -fr-stranded’. Briefly, raw counts were imported and filtered to remove genes with low or no expression (less than two counts per million in all experimental groups). Differential expression was calculated using voom from limma package^69^, with genes having a Benjamini–Hochberg false discovery rate (FDR) less than 0.05 and fold change greater than 1.5 being considered significant (unless otherwise indicated). Data were visualized using ggplot2 (ver. 3.0.0).

## Supplementary Information

Supplementary Information 1.

Supplementary Information 2.

Supplementary Information 3.

Supplementary Information 4.

## Data Availability

The datasets generated and analyzed during the current study are available in the European Nucleotide Archive (ENA) repository with the accession code PRJEB55015.
