# Supplementary material for: Parallel recovery of chromatin accessibility and gene expression dynamics from frozen human regulatory T cells
Source: Sci Rep. 2023 Apr 4;13:5506. doi: 10.1038/s41598-023-32256-6 (PMC10073253; doi:10.1038/s41598-023-32256-6)
Supplement: Supplementary file 1 — Supplementary Information 1. [file 41598_2023_32256_MOESM1_ESM.pdf]

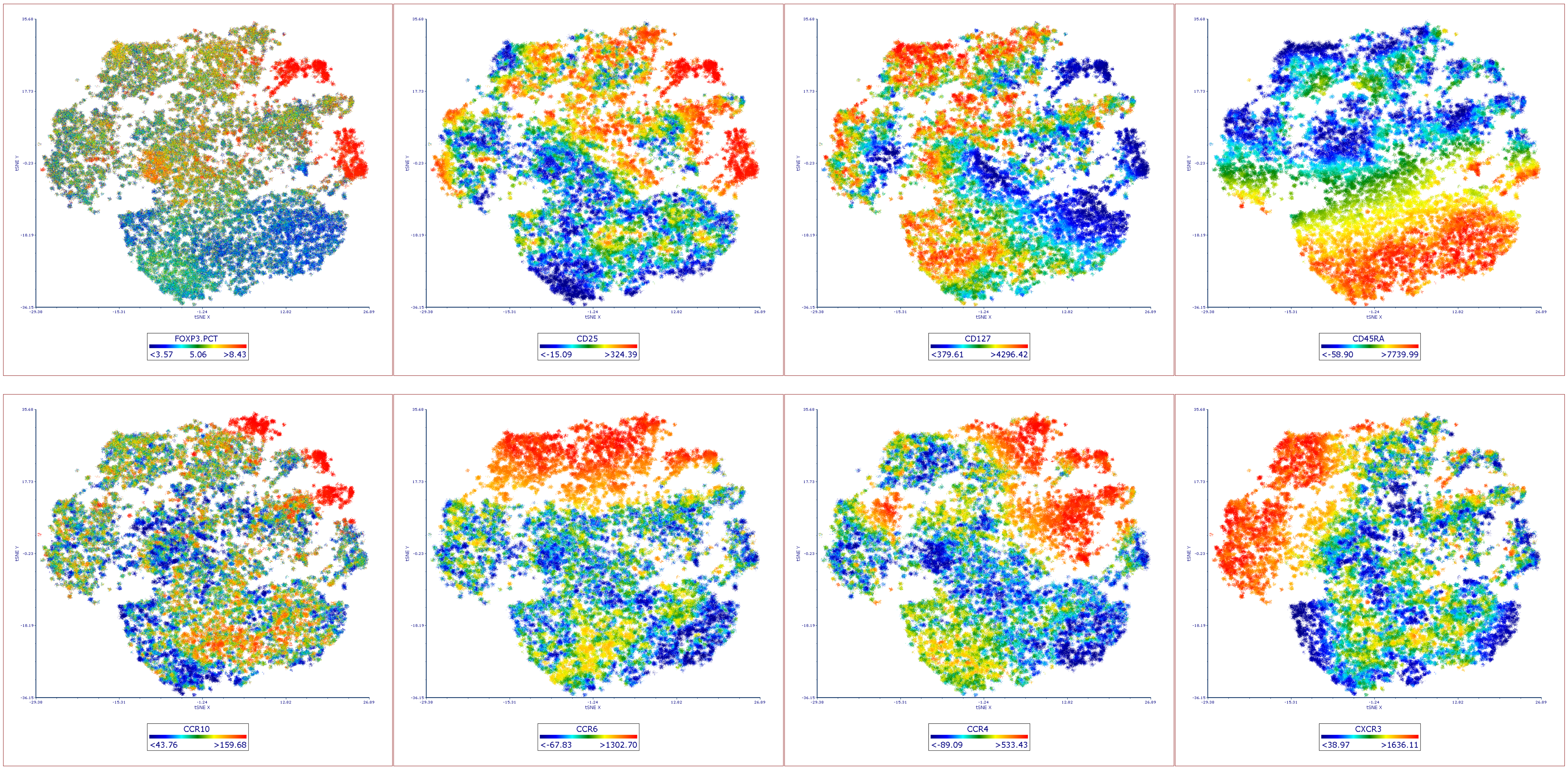

Supplementary Fig. 1 | Fluorescent markers projected across the t-SNE distribution with manual gating (FOXP3, CD25, CD127, CD45RA, CCR10, CCR6, CCR4 and CXCR3) for the concatenated fresh and thawed data.

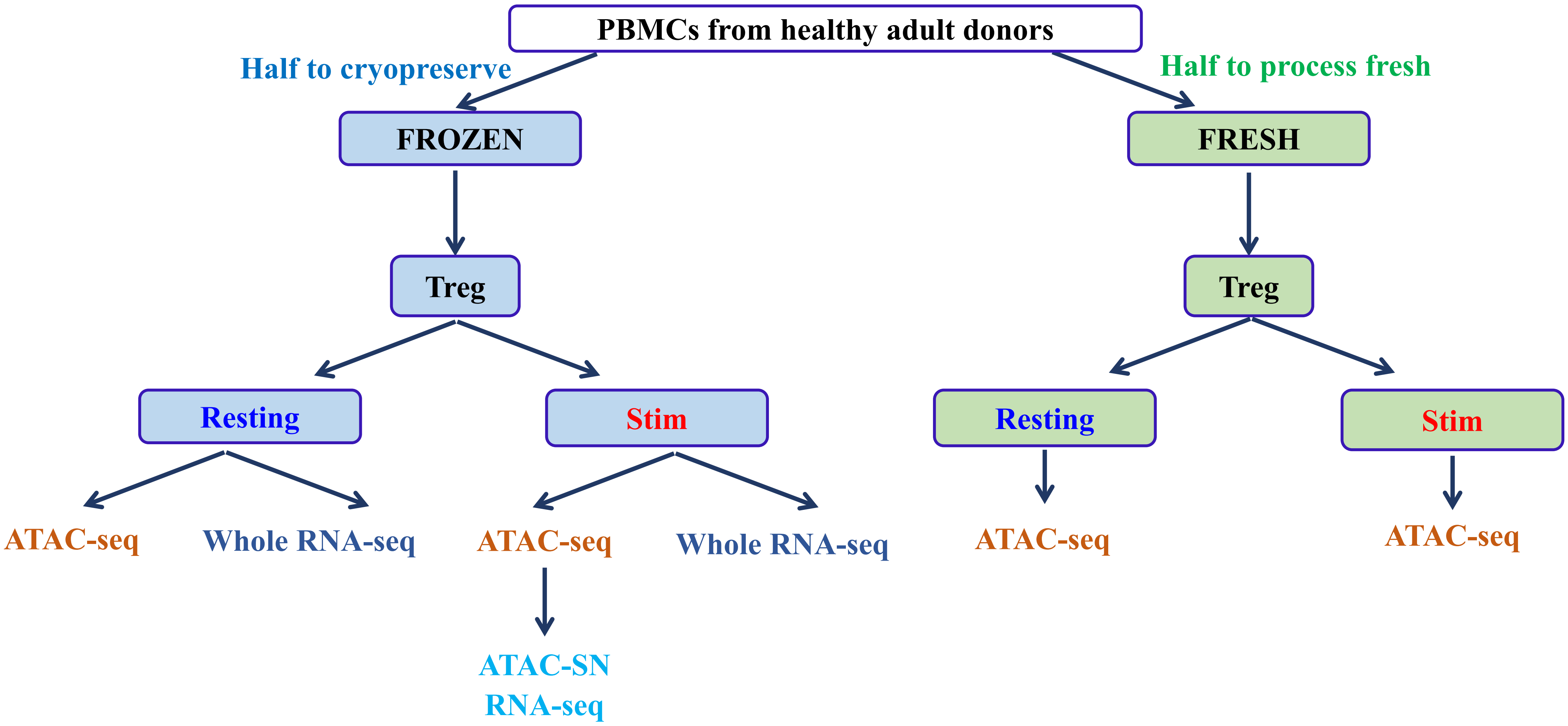

Supplementary Fig. 2 | Workflow from blood collection to ATAC-seq and RNA-seq library preparation.

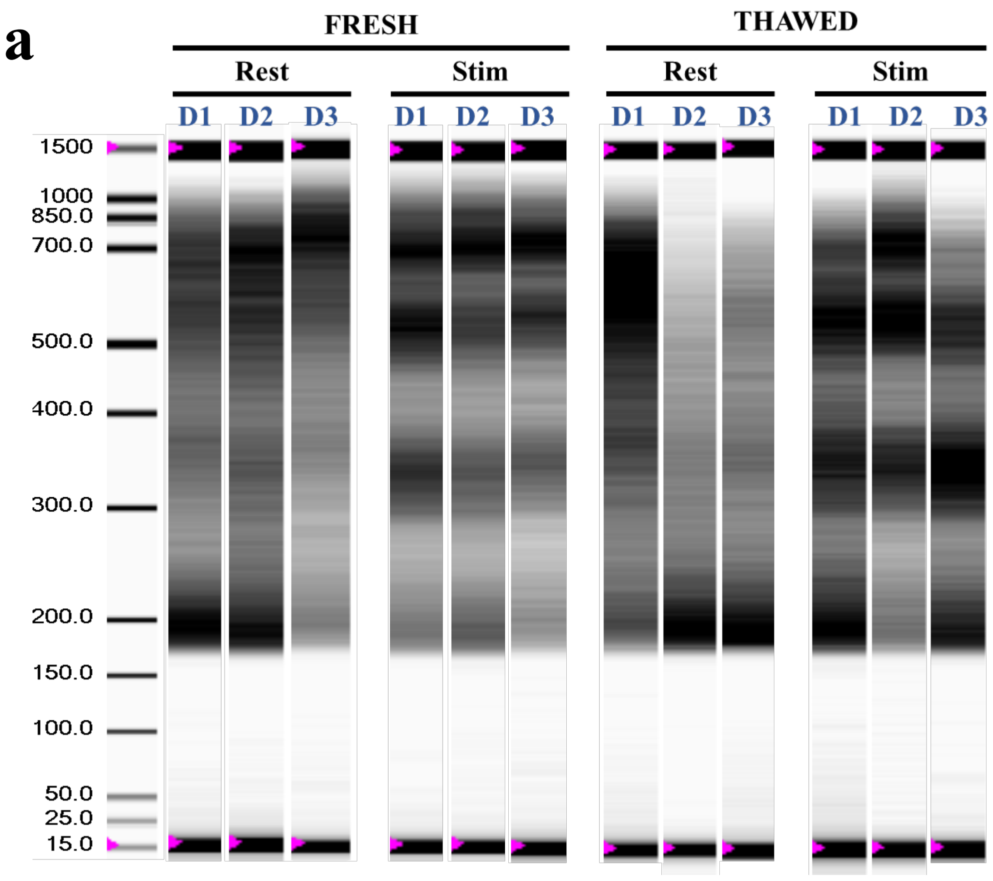

Supplementary Figure. 3 | Virtual gel demonstrating fragment size distribution of amplified fresh and thawed ATAC-seq libraries generated from resting or stimulated Treg cells.

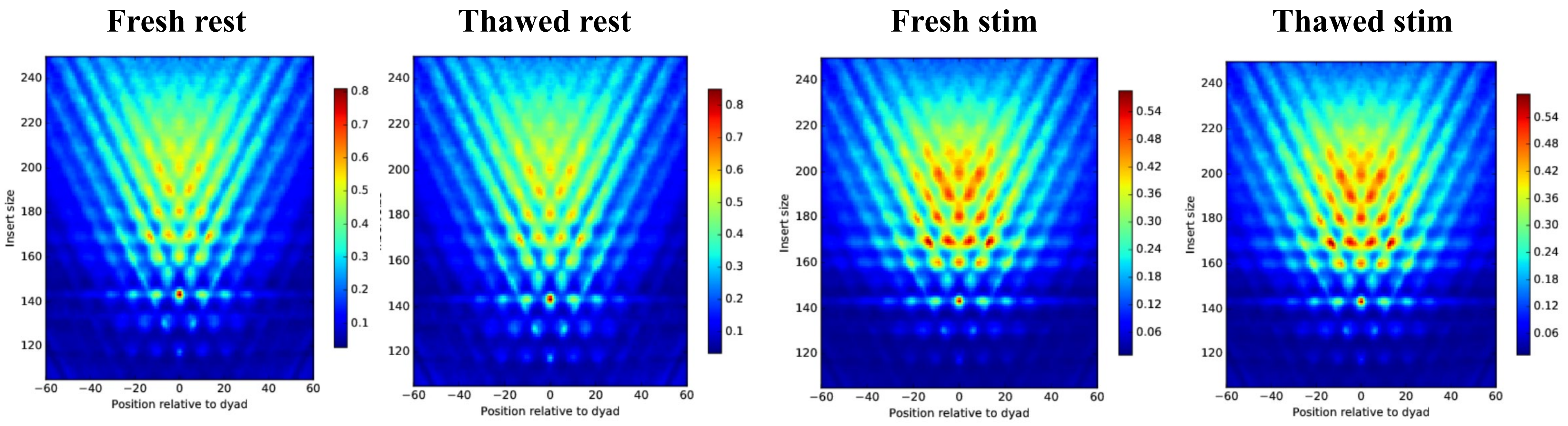

Supplementary Figure. 4 | Biobanking demonstrate structured ATAC-seq signal around nucleosomes. The V-plot maps the density of fragment sizes vs fragment midpoint position relative to nucleosome called by chemical mapping. A highly structured V-pattern observed at fragment sizes spanning a nucleosome for both fresh and thawed Treg cells. 2D nucleosomal “fingerprint” showing ATAC-seq signal around nucleosomes for fresh and thawed Treg cells during resting and stimulated state. V-plot, generated by nucleosome-positioning algorithm, NucleoATAC by Schep, Buenrostro (2015) maps the density of fragment sizes vs fragment midpoint positions relative to nucleosome dyads called by chemical mapping. Y-axis value represents insert size of fragments (bp) and X-axis value represents distance of the fragment midpoint from nucleosomes (bp). These aggregate protection profiles depict a V-shaped structure, where the apex of the “V” represents the smallest possible fragment that spans the DNA protected by a nucleosome. The calculation was performed on pooled sequencing reads representative of 3 adult donors.

a

Sample	Number of reproducible peaks
Fresh (resting)	41,587
Fresh (stimulated)	88,631
Thawed (resting)	45,534
Thawed (stimulated)	95,670

b

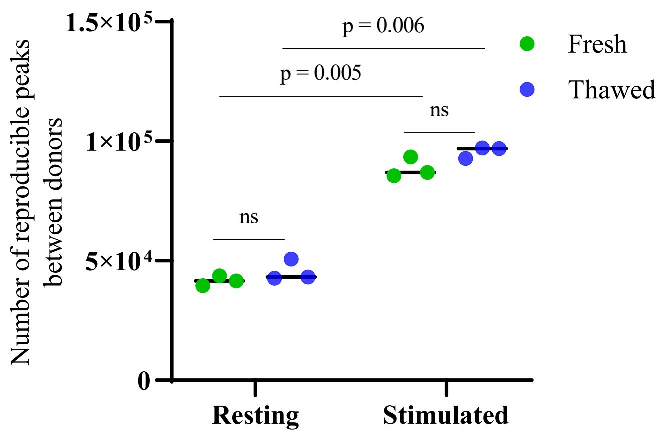

Supplementary Figure. 5 | Number of reproducible ATAC-seq peaks of donors determined using the Irreproducible Discovery Rate (IDR) method. Number of reproducible ATAC-seq peaks between donors were represented as (a) average of 3 donor-pair comparisons in each condition or (b) individual donor-pair comparison (donor 1 vs 2; donor 2 vs 3; donor 1 vs 3). Statistical significance of the difference is computed by Paired t-Test.

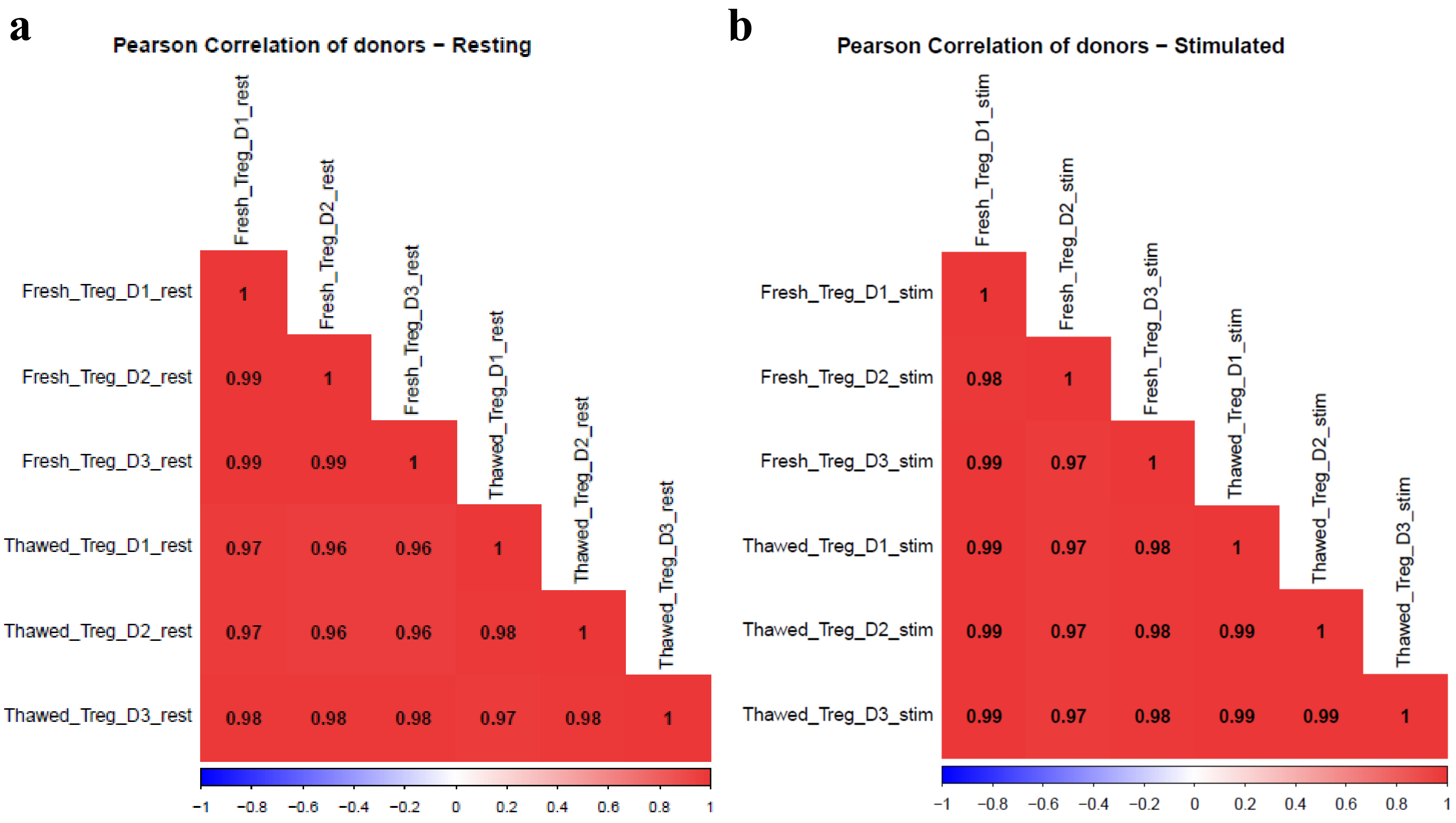

Supplementary Figure. 6 | Pearson correlation of ATAC-seq peaks for different donors in (a) **resting and** (b) **stimulation conditions.** Peaks are measured by count per million (CPM) of sequencing reads.

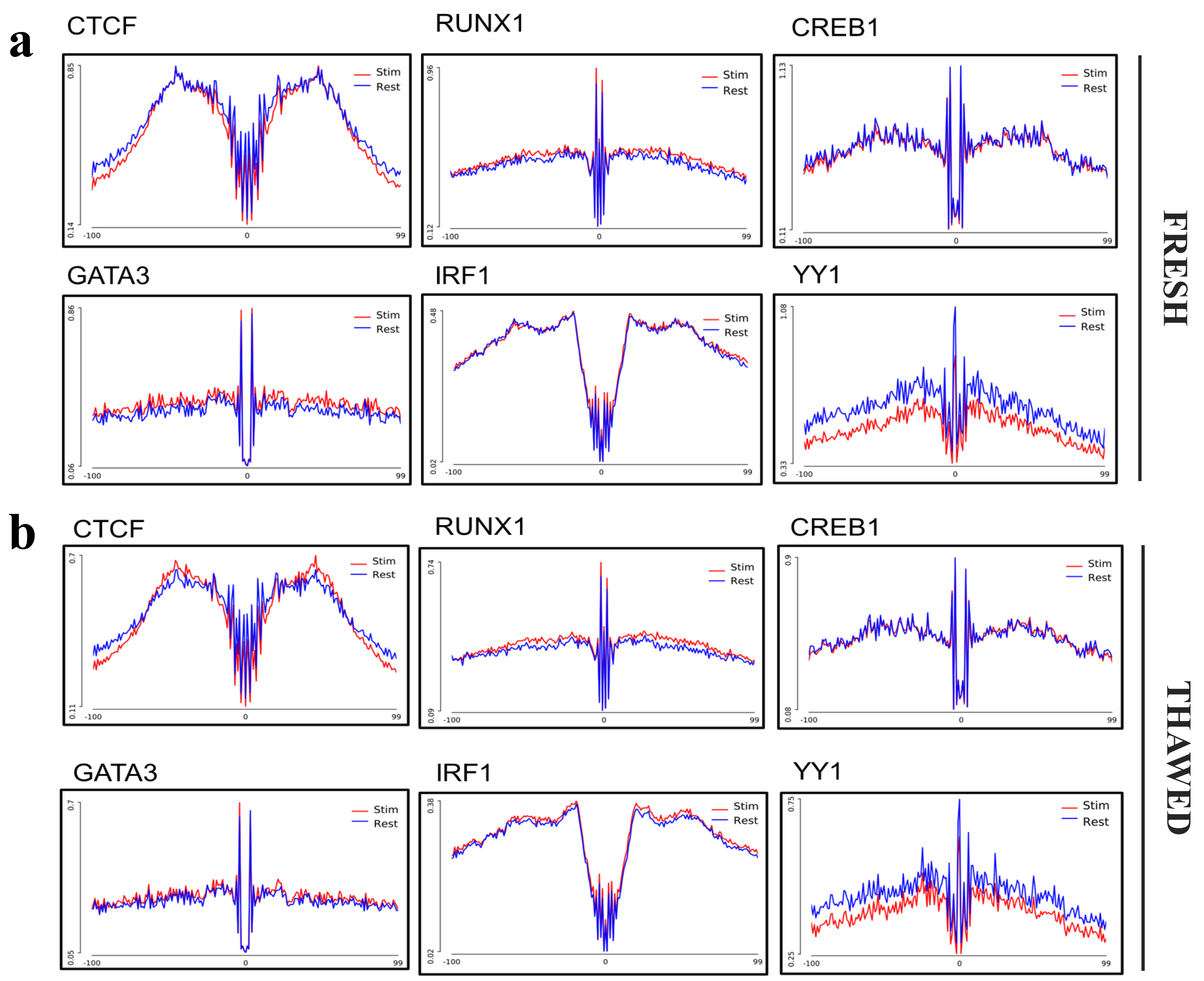

Supplementary Figure. 7 | Thawed Treg cells (b) **demonstrate similar responsiveness to stimulation as fresh cells** (a).

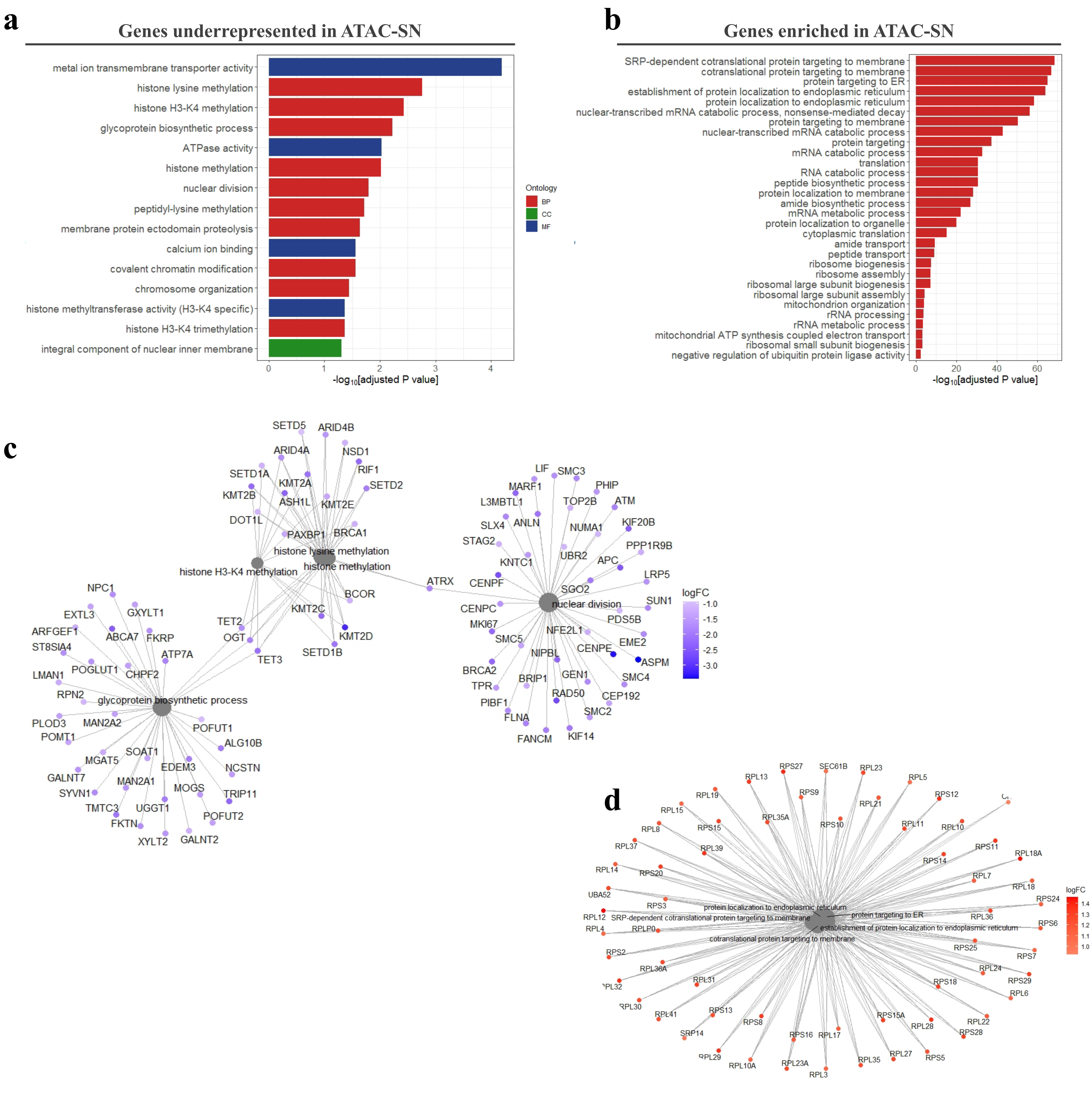

Supplementary Figure. 8 | Differentially expressed genes between ATAC-seq supernatant fraction (ATAC-SN) and whole cells are restricted by subcellular localization mechanism. Gene Ontology (GO) enrichment analyses showing top 30 significantly enriched GO terms associated with genes under-represented (a) or enriched (b) in ATAC-SN. Enrichment plots showing the most significant GO terms and the associated under-represented (c) or enriched (d) genes in ATAC-SN. All GO analyses were performed using a Bonferroni-adjusted p-value < 0.05 as the criteria for significance.

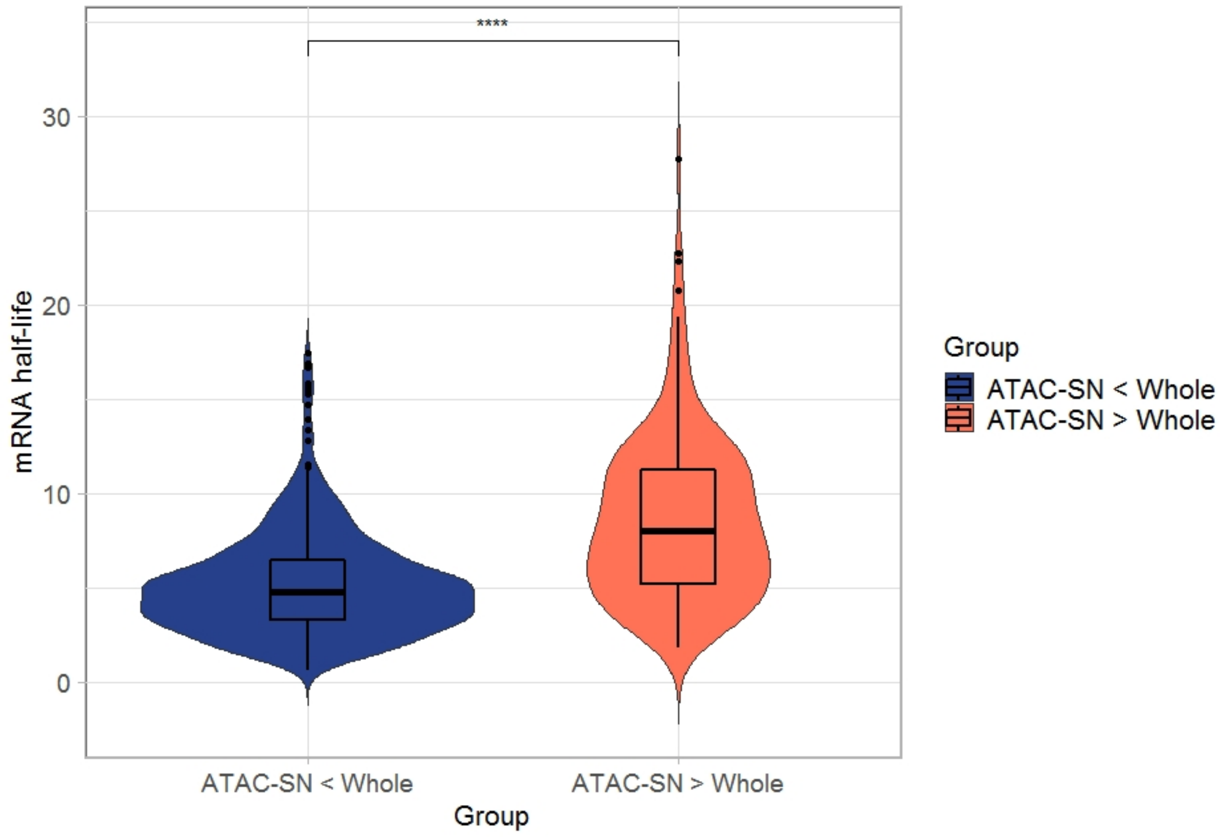

Supplementary Figure. 9 | mRNA half-life of differentially expressed genes between ATAC-SN and whole cell fractions. mRNA half-life data was determined from human HapMap lymphoblastoid cell lines (LCLs), measured as the ratio of nascent RNAs and total RNAs using two-hour 4-thiouridine (4sU) labeling (Duan et al., 2013).

ATAC-SN < Whole, genes detected at a lower level in ATAC-SN than whole cells; ATAC-SN > Whole, genes detected at a higher level in ATAC-SN than whole cells.

a

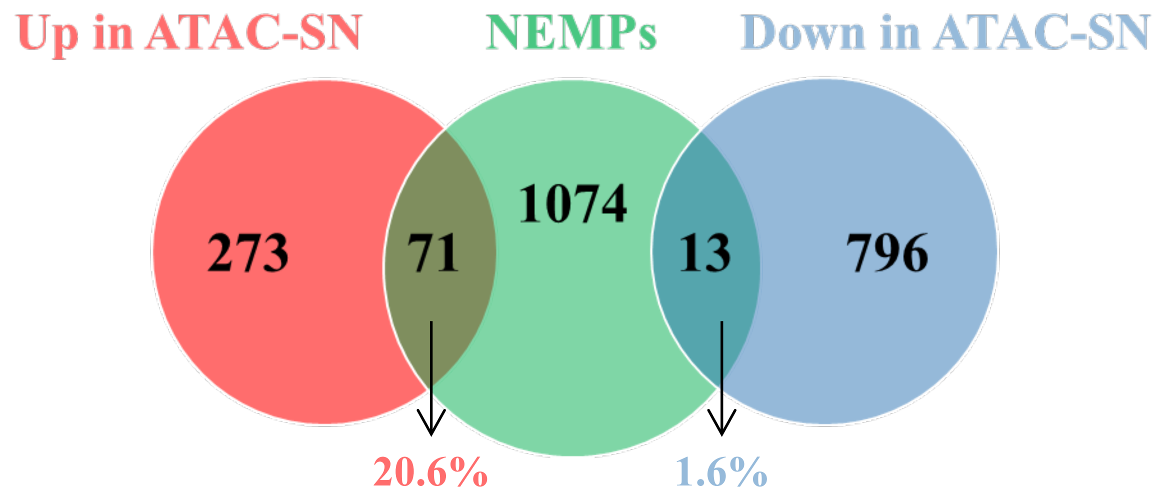

b

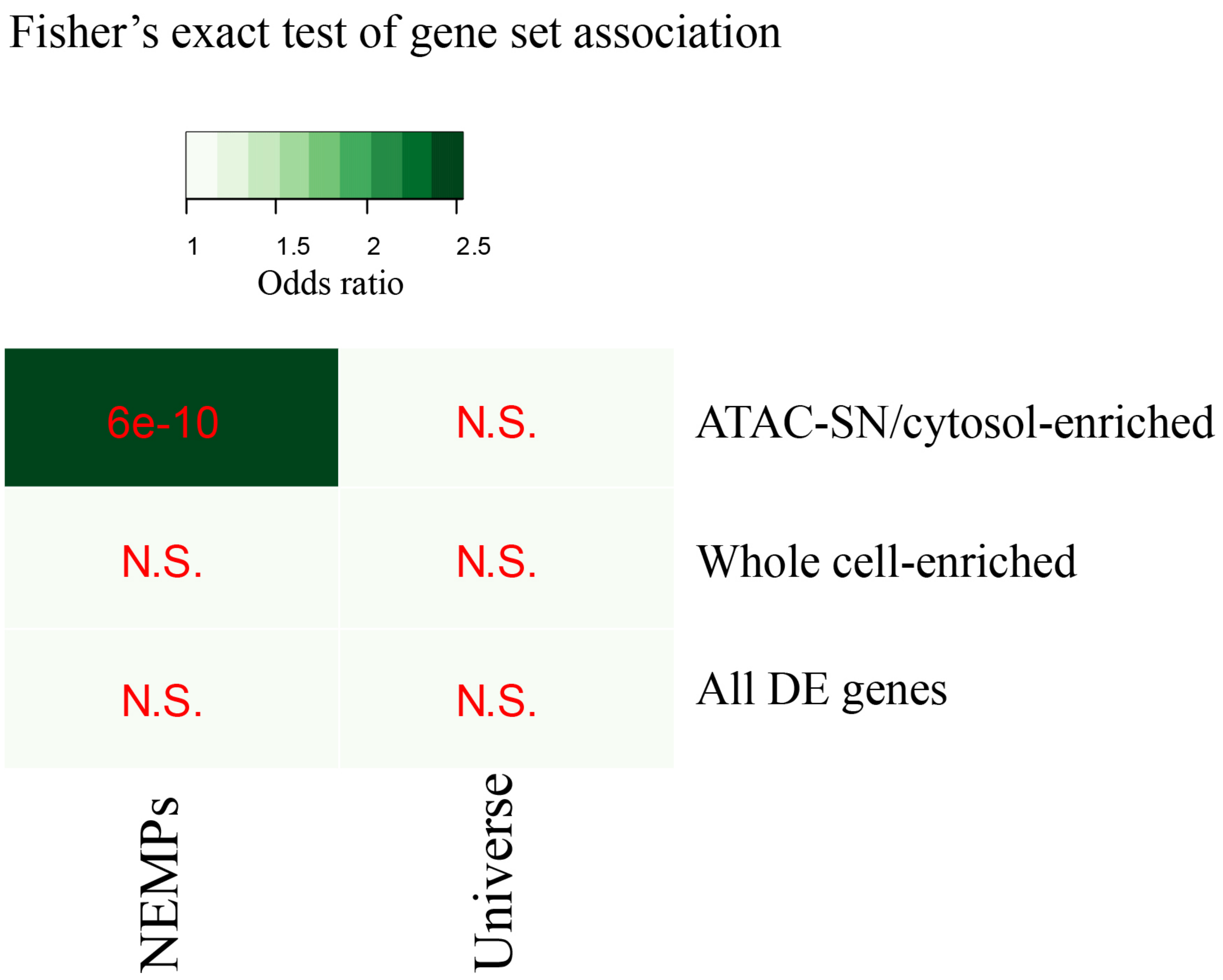

Supplementary Figure. 10 | Gene overlap (a) and Fisher's exact test (b) for assessing the strength of enrichment between genes up- or down-regulated in the ATAC-SN/cytosol fractions and human NEMPs (Nuclear-encoded-mitochondrial proteins). NEMPs database was obtained from the Broad Institute's human MitoCarta2.0 (Calvo et al 2015). These genes (n=1158) encode proteins with evidence for mitochondrial localization. The heatmap color scale represents the odds ratios and the significant p-values, computed from Fisher's exact test, are superimposed on the grids. All DE gene set represents all significantly differentially expressed genes between ATAC-SN and whole cell RNA-seq. Universe gene set contains all genes (DE and non-DE genes) detected in the RNA-seq dataset.

N.S., not significant; DE, differentially expressed.

	In-house Omni ATAC-seq ($CD4^+$ $CD25^{lo}$ $CD127^{hi}$)	Omni ATAC-seq ($CD3^+$ $CD4^+$ $CD45^+$)	Standard ATAC-seq (bulk $CD4^+$ T cells)
Overall alignment rate (%)	95.4	79.4	98.4
Proportion of uniquely mapped reads (%)	73.0	66.3	58.0
Library complexity (%)	91.2	83.5	70

Supplementary Figure. 11 | Assessment of read alignment and library complexity between different ATAC-seq methods. Datasets were generated using primary human T cells. Omni ATAC-seq datasets of $CD4^+$ $CD25^{lo}$ $CD127^{hi}$ were generated in-house (n=3 technical replicates) whereas raw sequencing datasets of $CD3^+$ $CD4^+$ $CD45^+$ and bulk $CD4^+$ populations were obtained from Corces et al. 2017 and Henriksson et al. 2017, respectively and processed using the same analysis pipeline.
